# Effects of Water and Nitrogen on Growth, Rhizosphere Environment, and Microbial Community of *Sophora alopecuroides*: Their Interrelationship

**DOI:** 10.3390/plants13141970

**Published:** 2024-07-18

**Authors:** Xiang Huang, Panxin Niu, Yude Gao, Wenwen Rong, Cunkai Luo, Xingxin Zhang, Ping Jiang, Mei Wang, Guangming Chu

**Affiliations:** 1 Agricultural College, Shihezi University, Shihezi 832003, China; huangxiang@stu.shzu.edu.cn (X.H.); panxinniu@163.com (P.N.); 20212012049@stu.shzu.edu.cn (W.R.); lck444973413@163.com (C.L.); shzujp@163.com (P.J.); 2 Practice Forest Farm, Xinjiang Agricultural University, Urumqi 830052, China; ydegao@163.com; 3 College of Grassland Science, Xinjiang Agricultural University, Urumqi 830052, China; zhangxingxinshz@163.com

**Keywords:** *Sophora alopecuroides*, water and nitrogen regulation, plant growth, rhizosphere soil environment, rhizosphere microbiota

## Abstract

The effective management of water and nitrogen is crucial in the artificial cultivation of medicinal plants. *Sophora alopecuroides*, a perennial herbaceous plant in the Fabaceae family, is extensively used in medicine, with alkaloids as its primary bioactive constituents. Nevertheless, there remains a significant knowledge gap regarding how rhizospheric microbial communities respond to varying water and nitrogen conditions and their intricate relationships with soil environments and the growth of *S. alopecuroides*. In this study, two-year-old *S. alopecuroides* were used in a two-factor, three-level water-nitrogen interaction experiment. The irrigation levels included W1 (30–35% of maximum water holding capacity), W2 (50–55%), and W3 (70–75%), while nitrogen levels comprised N1 (32 mg/kg), N2 (64 mg/kg), and N3 (128 mg/kg). The study assessed plant growth indicators, total alkaloid content, and rhizospheric soil physicochemical parameters of *S. alopecuroides*. High-throughput sequencing (16S rRNA and ITS) was employed to analyze variations in rhizospheric microbial community composition and structure. The results showed that Proteobacteria and Ascomycota are the predominant bacterial and fungal phyla in the rhizosphere microbial community of *S. alopecuroides*. The highest biomass and alkaloid accumulation of *S. alopecuroides* were observed under the N1W3 treatment (50% nitrogen application and 70–75% of maximum water holding capacity). Specifically, six bacterial genus-level biomarkers (*TRA3_20*, *MND1*, *env_OPS_17*, *SBR1031*, *Haliangium*, *S0134_terrestrial_group*) and six fungal genus-level biomarkers (*Pseudeurotium*, *Rhizophagus*, *Patinella*, *Pseudeurotium*, *Patinella*, *Rhizophagus*) were identified under the N1W3 treatment condition. In the partial least squares path modeling (PLS-PM), water and nitrogen treatments demonstrated markedly positive direct effects on soil physicochemical parameters (*p* < 0.01), while showing significant negative direct impacts on alkaloid accumulation and plant growth indicators (*p* < 0.05). Soil physicochemical parameters, in turn, significantly negatively affected the rhizosphere fungal community (*p* < 0.05). Additionally, the rhizosphere fungal community exhibited highly significant negative direct effects on both the plant growth indicators and total alkaloid content of *S. alopecuroides* (*p* < 0.01). This study provides new insights into the interactions among rhizosphere soil environment, rhizosphere microbiota, plant growth, and alkaloid accumulation under water and nitrogen regulation, offering a scientific basis for the water and nitrogen management in the cultivation of *S. alopecuroides*.

## 1. Introduction

*Sophora alopecuroides* L., a perennial herb belonging to the genus *Sophora* in the Fabaceae family, is widely distributed across dry and semi-arid regions of the Asian continent [1,2]. In China, it is predominantly found in northwest deserts and semi-deserts such as Xinjiang, Inner Mongolia, Gansu, and Ningxia [3]. *S. alopecuroides* exhibits strong abilities in drought resistance, wind and sand tolerance, and adaptation to nutrient-poor soils. It is an important plant for windbreak, sand fixation, and soil conservation, playing a significant role in desertification recovery and possessing crucial ecological value [4]. Furthermore, the entire plant of *S. alopecuroides* is utilized in traditional medicine, biopesticides, organic fertilizers, and feed additives, highlighting its economic importance [5]. Alkaloids, the primary active constituents of *S. alopecuroides*, exhibit notable pharmacological activities such as anti-inflammatory [6], antimicrobial [7], and anticancer properties [8]. Currently, *S. alopecuroides* is primarily supplied to the market through wild resources, with an annual market demand reaching several tens of thousands of tons, indicating a significant supply gap. Artificial domestication and cultivation have gradually become the main methods of *S. alopecuroides* production.

Irrigation and fertilization are the most critical agronomic practices in the artificial cultivation of medicinal plants [9]. Inefficient irrigation and fertilization methods can lead to a reduction in the quality of medicinal plants, wastage of water resources, and significant ecological damage [10]. Nitrogen is not only an essential nutrient for the growth and development of medicinal plants but also a crucial factor influencing the accumulation of their secondary metabolites. Additionally, adequate water availability enhances the transport capacity of nitrogen within the plant [11]. Li et al. [12] found that the coupling of water and nitrogen significantly affects the total flavonoid and chlorogenic acid contents in *Chuzhou Chrysanthemum morifolium*, with a notable interaction between water and nitrogen. The impact of nitrogen fertilizer on the total flavonoid and chlorogenic acid contents was greater than that of water availability. Moreover, Taha et al. [13] demonstrated that irrigation significantly affects the number of seeds per plant, biomass, and grain yield of Ajowan, with essential oil yield notably increasing with higher nitrogen levels. However, there is a threshold effect of water and nitrogen regulation on the production of medicinal plants; once this threshold is exceeded, additional inputs of water and nitrogen do not yield significant increases in yield [14]. Therefore, moderate regulation of water and nitrogen is crucial for enhancing both the growth and quality of medicinal plants.

The rhizosphere, one of the most active interfaces of microbial activity in the soil surrounding plant roots, is a critical site for plant-soil-microbe interactions and the exchange of information and substances [15]. Rhizosphere microorganisms are often considered a second plant genome [16]. The community structure and diversity of these microorganisms are influenced by soil environmental conditions, with key driving factors being soil physicochemical parameters such as nutrients, moisture content, organic matter, and pH [17]. Different water and nitrogen management practices directly influence the rhizospheric soil environment, potentially inducing significant changes in rhizosphere microbial communities. Excessive or insufficient nitrogen application and irrigation can both hinder the establishment of optimal rhizosphere microbial communities in plants. Rhizosphere microorganisms play diverse roles in the growth, development, metabolism, and accumulation of active compounds in medicinal plants [18]. However, the effects of water and nitrogen regulation on rhizosphere microbial diversity and activity in medicinal plants are complex. While high-throughput sequencing technologies have advanced our understanding of how individual factors impact rhizosphere microbial communities [19,20], comprehensive knowledge of their interactive effects remains limited.

Previous studies have investigated the agronomic traits, biomass, and alkaloid accumulation of *S. alopecuroides* under varying water and nitrogen regimes [21,22,23]. However, several critical scientific questions remain unclear: What are the specific impacts of water and nitrogen regulation on the growth of *S. alopecuroides* and the rhizospheric soil environment, and how do these factors interact? Therefore, this study utilized the Illumina Novaseq 6000 platform for high-throughput sequencing of rhizosphere microbiota under different water and nitrogen conditions in *S. alopecuroides*. Integrated with the growth indicators and rhizospheric soil physicochemical parameters of *S. alopecuroides*, our research aims to investigate the effects of varying water and nitrogen conditions on: (1) the growth and rhizospheric soil physicochemical parameters of *S. alopecuroides*; (2) the composition and diversity of rhizosphere microbial communities associated with *S. alopecuroides*; and (3) the interrelationships among soil physicochemical parameters, rhizosphere microbiota, and the growth of *S. alopecuroides*. Based on previous findings, we hypothesize that adequate water irrigation and low nitrogen conditions will promote the establishment of a favorable rhizospheric environment for *S. alopecuroides*, thereby enhancing its growth. This study aims to provide a scientific basis for the management of water and nitrogen in the cultivation of *S. alopecuroides*, offering new insights into the complex interactions among soil environment, rhizosphere microbiota, and medicinal plant growth.

## 2. Results

### 2.1. Plant Growth and Rhizospheric Soil Physicochemical Parameters

**Figure 1:**
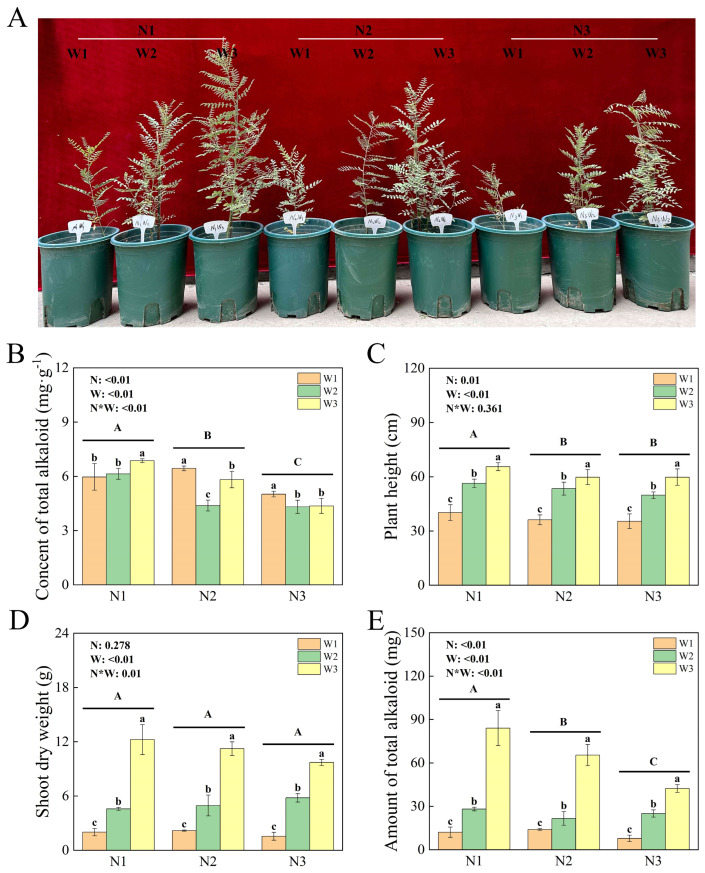
Growth of *Sophora alopecuroides* under different water and nitrogen treatments. Plant phenotype (**A**), content of total alkaloid (**B**), plant height (**C**), Shoot dry weight (**D**), amount of total alkaloid (**E**) of *S. alopecuroides*.

The phenotypic responses of *S. alopecuroides* under different water-nitrogen coupling treatments are illustrated in Figure 1A. Significant differences (*p* < 0.01) were observed in the alkaloid content of *S. alopecuroides* among treatments influenced by nitrogen, water, and their interaction, with the highest alkaloid content recorded in the N1W3 treatment (Figure 1B). Nitrogen and water significantly affected the plant height of *S. alopecuroides* across treatments (*p* < 0.01), while the water-nitrogen interaction did not show significant differences among treatments. Furthermore, under different nitrogen levels, the plant height of *S. alopecuroides* exhibited an increasing trend with higher irrigation levels (Figure 1C). Water and the water-nitrogen interaction exhibited significant differences (*p* < 0.01) in the shoot dry weight of *S. alopecuroides* across treatments, whereas nitrogen levels did not show significant differences among treatments. Additionally, under different nitrogen levels, the shoot dry weight of *S. alopecuroides* increased with higher irrigation levels (Figure 1D). Nitrogen, water, and their interaction significantly influenced (*p* < 0.01) the amount of total alkaloid of *S. alopecuroides* across treatments, with the highest amount of total alkaloid observed in the N1W3 treatment (Figure 1E).

The soil physicochemical parameters of *S. alopecuroides* under different water and nitrogen treatments are presented in Figure S1. Nitrogen significantly affected TN, AP, pH, and SOM (*p* < 0.05). Water significantly influenced TN, AP, AK, and SEC (*p* < 0.05). The interaction between water and nitrogen significantly impacted TN, AN, AP, pH, SEC, and SOM (*p* < 0.05). At different nitrogen levels, TN and AP content were significantly higher at the N3 level compared to the N1 and N2 levels (*p* < 0.05). Soil pH and SOM content were significantly higher at the N2 level compared to the N1 and N3 levels (*p* < 0.05). There were no significant differences in AN, AK, and SEC across different nitrogen levels.

### 2.2. Overview of Sequencing Data

A total of 9,634,656 raw reads (3,017,748 16S + 6,366,671 ITS) were identified across 27 soil samples. The number of raw reads per sample ranged from 91,518 to 132,086 for 16S and from 178,379 to 344,783 for ITS. After sequence quality filtering, denoising, and merging, the proportion of high-quality reads retained for downstream analyses ranged from 85.15% to 92.63% for 16S and from 89.63% to 97.93% for ITS (Table S2). To mitigate the variation in read numbers across different samples, we randomly selected 78,438 reads per sample for 16S and 169,934 reads per sample for ITS. Clustering resulted in a total of 48,650 bacterial ASVs and 10,336 fungal ASVs. The corresponding rarefaction curves for the Chao1 index and observed_features index tended to plateau as sequencing depth increased (Figure S2), and the goods_coverage index exceeded 99% for all samples, indicating that the sequencing depth in this study was sufficient.

### 2.3. Composition of Bacterial and Fungal Communities

**Figure 2:**
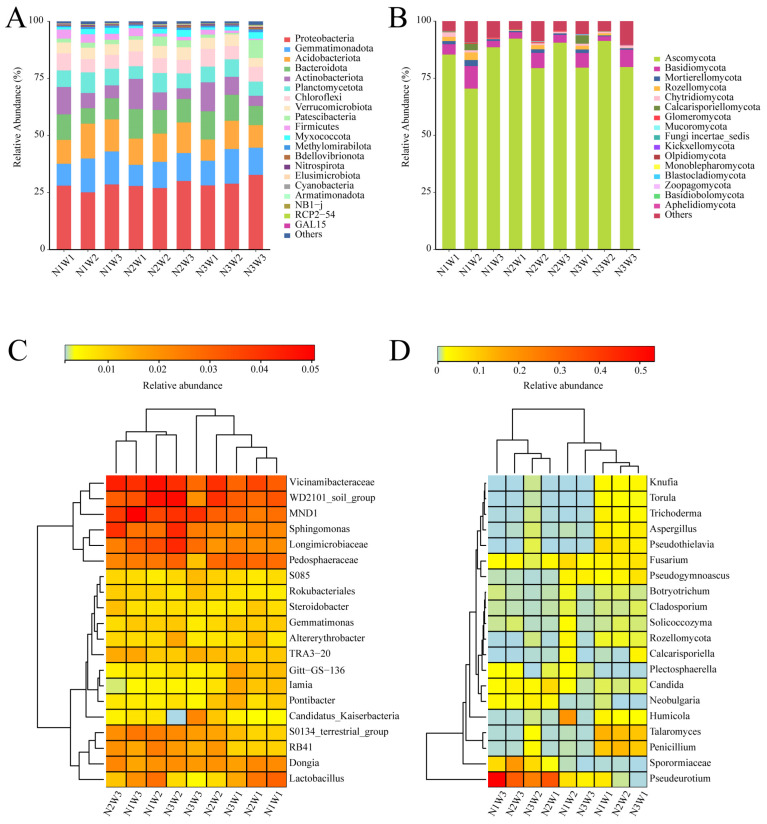
Rhizosphere microbes of *S. alopecuroides* under different water and nitrogen treatments. Bacterial composition at the phylum level in the rhizosphere (**A**), fungal composition at the phylum level in the rhizosphere (**B**), relative abundance heatmap of the top 20 bacterial genera (**C**) and the top 20 fungal genera (**D**) in the rhizosphere.

A total of 42 phyla, 123 classes, 316 orders, 482 families, 863 genera, and 401 species of bacteria, as well as 16 phyla, 51 classes, 120 orders, 309 families, 633 genera, and 1036 species of fungi, were detected across all samples (Table S3). This study examined the taxonomic composition of microorganisms in each treatment at the phylum and genus levels. Most bacteria and fungi were common across the nine treatments at both the phylum and genus levels (Figure S3). At the phylum level, Proteobacteria was the most dominant bacterial phylum, with relative abundance ranging from 25.13% to 32.78% (Figure 2A), while Ascomycota was the most dominant fungal phylum, with relative abundance ranging from 70.49% to 92.38% (Figure 2B). At the genus level, the relative abundances of six bacterial genera (*Vicinamibacteraceae*, *WD2101_soil_group*, *MND1*, *Sphingomonas*, *Longimicrobiaceae*, *Pedosphaeraceae*) were consistent across different treatment groups and were significantly higher than those of other genera (Figure 2C). Among fungi, *Pseudeurotium* was a genus with high relative abundance across different treatment groups (Figure 2D).

### 2.4. Alpha and Beta Diversity of Rhizosphere Microbiota

Multivariate analysis of variance revealed significant effects of nitrogen, water, and their interaction on the abundance of bacteria and fungi in each sample, as reflected by the observed_features, chao1, and Ace indices (*p* < 0.05) (Table S4). Additionally, the observed_features, chao1, and Ace indices were significantly higher in bacteria than in fungi across different treatments (Table S5). Water and the interaction between water and nitrogen also significantly influenced the diversity of bacteria and fungi in each sample, as indicated by the simpson index (*p* < 0.05). However, nitrogen did not significantly affect the diversity of bacteria and fungi in each sample, as reflected by the shannon and faith indices (*p* > 0.05) (Table S4).

**Figure 3:**
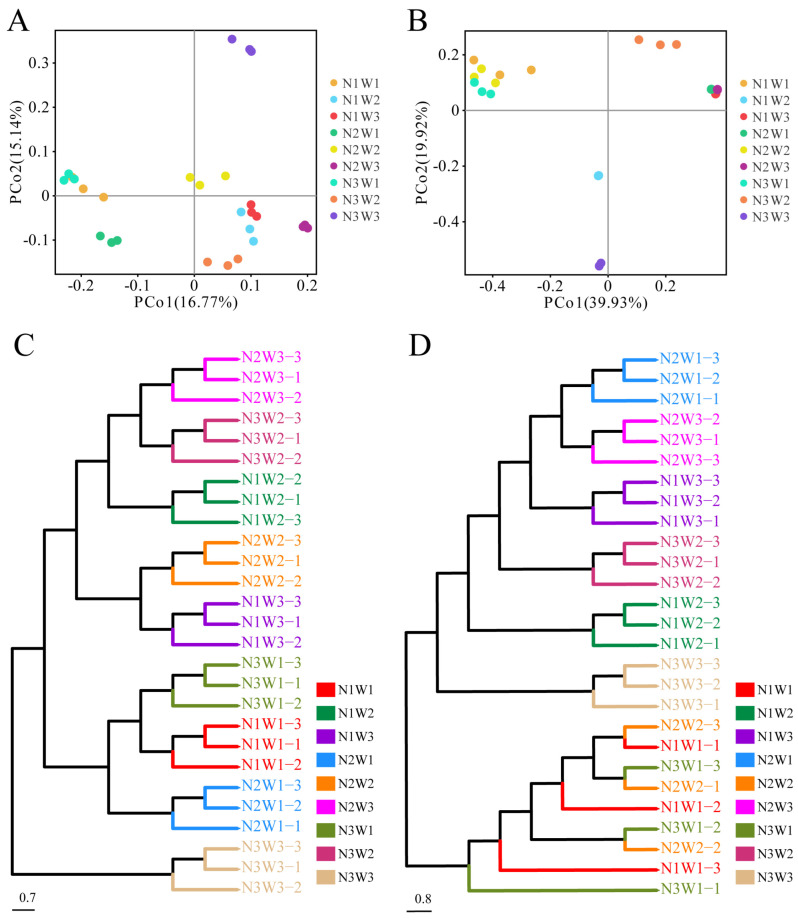
Alpha and beta diversity of rhizosphere microbiota in *S. alopecuroides* under different water and nitrogen treatments. PCoA analysis of bacteria (**A**) and fungi (**B**), UPGMA clustering analysis of bacteria (**C**) and fungi (**D**).

In this study, Beta diversity analysis was primarily based on the Bray-Curtis algorithm to compute distances between samples and obtain β values among them. Two-way ANOVA results indicated significant effects of water and the interaction between water and nitrogen on Beta diversity of bacteria and fungi in each sample (*p* < 0.05). Nitrogen did not significantly influence Beta diversity of bacteria in each sample (*p* > 0.05), but had a significant effect on fungi (*p* < 0.05). Anosim analysis of inter-group differences showed significant dissimilarities between bacterial (*R* = 0.973, *p* = 0.001) and fungal (*R* = 0.908, *p* = 0.001) communities among the 27 samples (Figure S4). PCoA analysis revealed distinct microbial community structures among the nine treatments. In bacteria, the two principal components (PCo1 and PCo2) explained 16.77% and 15.14% of the variation, respectively. Based on PCo1, the nine treatment groups were divided into two clusters, with N3W3 clearly separated from the other treatments along PCo2 (Figure 3A). Similarly, in fungi, the two principal components (PCo1 and PCo2) explained 39.93% and 19.92% of the variation, respectively. Based on PCo1, the nine treatment groups were divided into two clusters, with N3W1 and N3W3 distinctly separated from the other treatments along PCo2 (Figure 3B). Unweighted pair-group method with arithmetic mean (UPGMA) clustering analysis results based on Bray-Curtis distances between different treatment groups reflected the similarity of microbial community structures consistent with the PCoA analysis results (Figure 3C,D).

### 2.5. Analysis of Significantly Different Species

**Figure 4:**
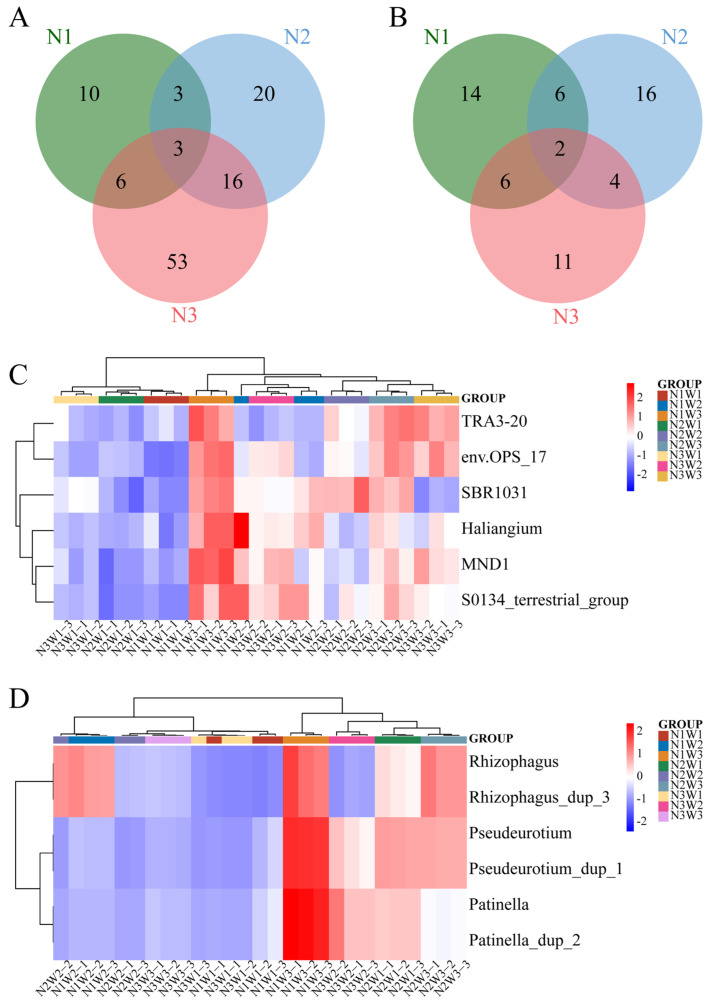
Analysis of significantly different species of *S. alopecuroides* under different water and nitrogen treatments. Venn diagrams analysis of bacteria (**A**) and fungi (**B**), and clustering of relative abundance for bacteria (**C**) and fungi (**D**).

Significantly different bacterial genera (*p* < 0.05) between the groups are listed in Table S6. There are 22 genera common to both N1W3 vs. N1W1 and N1W3 vs. N1W2 (N1), 42 genera common to both N2W3 vs. N2W1 and N2W3 vs. N2W2 (N2), and 78 genera common to both N3W3 vs. N3W1 and N3W3 vs. N3W2 (N3). The Venn diagram further shows that among these common genera, 10 are unique to N1 (*TK10*, *Massilia*, *Devosia*, *Asticcacaulis*, *Noviherbaspirillum*, *Prosthecobacter*, *TX1A-33*, *Constrictibacter*, *Roseimicrobium*, *Methyloligellaceae*), and 3 genera (*TRA3-20*, *0319-7L14*, *Pedobacter*) are common to N1, N2, and N3 (Figure 4A).

Significantly different fungal genera (*p* < 0.05) between the groups are listed in Table S7. There are 28 genera common to both N1W3 vs. N1W1 and N1W3 vs. N1W2 (N1), 28 genera common to both N2W3 vs. N2W1 and N2W3 vs. N2W2 (N2), and 23 genera common to both N3W3 vs. N3W1 and N3W3 vs. N3W2 (N3). The Venn diagram further shows that among these common genera, 14 are unique to N1 (*Talaromyces*, *Aspergillus*, *Pseudogymnoascus*, *Patinella*, *Lectera*, *Albifimbria*, *Emericellopsis*, *Setophoma*, *Sarocladium*, *Gymnostellatospora*, *Filobasidium*, *Halosphaeriaceae_gen_Incertae_sedis*, *Cystofilobasidium*, *Tetracladium*), and 2 genera (*Neobulgaria*, *Acremonium*) are common to N1, N2, and N3 (Figure 4B).

LEfSe analysis was further used to identify biomarkers with statistically significant differences between the groups. The LEfSe cladogram for bacteria is shown in Figure S5A, among them, 6, 15, and 21 biomarkers at the genus level were identified in N1W3, N2W3, and N3W3, respectively (*p*-value < 0.05, LDA score > 3) (Table S8). The LEfSe cladogram for fungi is shown in Figure S5B, among them, 6, 8, and 11 biomarkers at the genus level were identified in N1W3, N2W3, and N3W3, respectively (*p*-value < 0.05, LDA score > 3) (Table S9). The relative abundance clustering results further indicate that the abundance of these biomarkers in the N1W3 treatment group was significantly higher than in the other treatment groups for both bacteria and fungi (Figure 4C,D).

### 2.6. Mantel Tests and Integrated Analysis Based on PLS-PM

**Figure 5:**
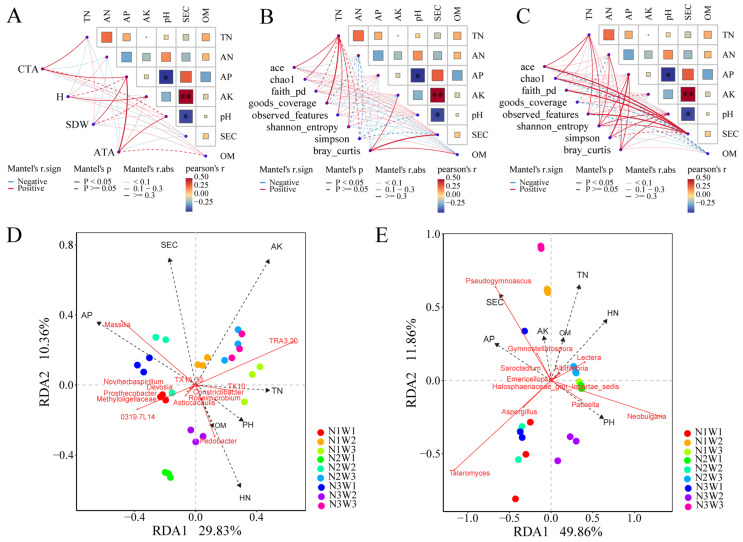
Mantel test and redundancy analysis (RDA). Mantel test of plant growth indices (**A**), rhizosphere bacterial diversity (**B**), and rhizosphere fungal diversity (**C**) with soil physicochemical parameters. At the genus level, rhizosphere bacteria (**D**) and rhizosphere fungi (**E**) showing significant differences between different treatment groups were subjected to RDA analysis with soil physicochemical parameters. *: significant at the 0.05 level, **: significant at the 0.01 level.

Mantel tests were conducted to assess relationships among soil physicochemical parameters, richness and diversity of rhizosphere microbial communities, plant growth indices, and total alkaloid content (Figure 5). AP and SEC were significantly negatively correlated with pH (*p* < 0.05), while AK showed a highly significant positive correlation with SEC (*p* < 0.05). The total alkaloid content showed significant positive correlations with TN and AP (*p* < 0.05). Plant height and shoot dry weight were significantly positively correlated with AK and AP, respectively (*p* < 0.05). Additionally, the amount of total alkaloid exhibited significant positive correlations with AP and pH (*p* < 0.05) (Figure 5A). TN showed significant positive correlations with bacterial and fungal community richness (ace, chao1, observed_features) and diversity (shannon_entropy) (*p* < 0.05) (Figure 5B,C). TN, AN, AP, and pH were all significantly positively correlated with fungal community diversity (shannon_entropy) (*p* < 0.05). Additionally, SEC exhibited significant positive correlations with both bacterial community richness and diversity (ace, chao1, observed_features, shannon_entropy, faith_pd) (*p* < 0.05) (Figure 5C). At the genus level, bacteria and fungi showing significant differences between different treatments were subjected to RDA analysis with soil physicochemical parameters. In bacteria, *TK10* and *TRA3-20* were positively correlated with TN, and *Pedobacter* showed a positive correlation with AN. Conversely, 0319-7L14 exhibited negative correlations with both TN and AN (Figure 5D). Among fungi, *Talaromyces* and *Aspergillus* were negatively correlated with TN and AN (Figure 5E).

**Figure 6:**
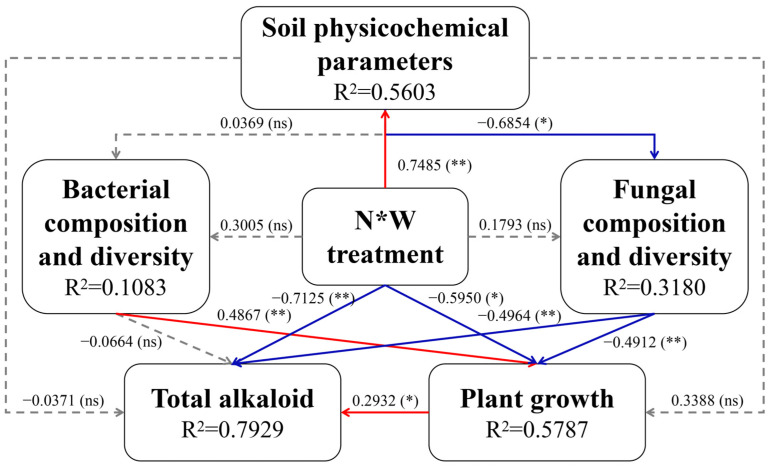
Partial least squares path modeling of soil physicochemical parameters, rhizosphere microbiota, and plant growth. *: significant correlation (*p* < 0.05), **: highly significant correlation (*p* < 0.01), ns: not correlated. The red line indicates a positive correlation, and the blue line indicates a negative correlation.

We further analyzed the interrelationships among soil physicochemical parameters, rhizosphere bacterial community richness and diversity, rhizosphere fungal community richness and diversity, plant growth indices, and total alkaloid content using PLS-PM analysis (Figure 6). Nitrogen and water treatments exerted highly significant positive effects on soil physicochemical parameters (*p* < 0.01), significant negative effects on total alkaloid content (*p* < 0.01), and significant negative effects on plant growth indices (*p* < 0.05), but did not significantly affect bacterial and fungal community richness and diversity. Soil physicochemical parameters had significant negative effects on fungal community richness and diversity (*p* < 0.05). Bacterial community richness and diversity had highly significant positive effects on plant growth (*p* < 0.01), while fungal community richness and diversity exerted highly significant negative effects on both plant growth and total alkaloid content (*p* < 0.01). In addition, plant growth had a significant positive effect on total alkaloid content (*p* < 0.05).

## 3. Discussion

Nitrogen and water regulation play a crucial role in the cultivation and management of medicinal plants, significantly affecting plant growth, biomass accumulation, and the content and quality of medicinal components [24]. This study found that under different nitrogen levels, the height and shoot dry weight of *S. alopecuroides* increased with higher irrigation levels, indicating that an appropriate increase in irrigation can significantly promote growth and development. This finding is consistent with previous studies showing that sufficient water supply can significantly enhance growth rate and biomass accumulation of medicinal plants [25]. Previous studies have shown that sufficient water supply can promote the absorption of nitrogen in plants, thereby increasing the synthesis rate of nitrogen-containing secondary metabolites [26,27]. In this study, the total alkaloid content in the shoot parts of *S. alopecuroides* was highest in the N1W3 treatment. This indicates that under low nitrogen conditions, sufficient irrigation significantly enhanced the absorption of nitrogen, leading to an increase in total alkaloid content. Furthermore, significant differences were observed in the amount of total alkaloid of *S. alopecuroides* due to the effects of nitrogen, water, and their interaction. This underscores the importance of nitrogen and water management in the cultivation of *S. alopecuroides*, suggesting that appropriate low nitrogen treatment combined with sufficient irrigation can optimize production and accumulation of medicinal components.

The dual-factor regulation of water and nitrogen significantly affects soil physicochemical parameters, exhibiting synergistic effects that differ from the effects of water or nitrogen regulation alone [28]. In this study, the interaction between water and nitrogen was significantly correlated with more soil physicochemical parameters compared to individual water or nitrogen regulation, indicating that the combined effect of water and nitrogen on the rhizosphere soil of *S. alopecuroides* is more complex. The soil TN content was significantly higher at the N3 level compared to the N1 and N2 levels (*p* < 0.05), suggesting that the higher nitrogen application at the N3 level promotes nitrogen accumulation in the soil. This also accelerated the cycle of phosphorus, resulting in an increase in AP content in the rhizosphere soil of *S. alopecuroides*, which may be related to the increase of phosphatase activity in soil by high nitrogen [29]. Additionally, soil pH and SOM content were significantly higher at the N2 level than at the N1 and N3 levels (*p* < 0.05). This may be because the appropriate amount of nitrogen application at the N2 level avoids the acidification of the rhizosphere soil of *S. alopecuroides* and reduces the loss of soil organic matter due to the mineralization of residual inorganic nitrogen in the soil [30].

Water and nitrogen regulation profoundly influence the composition and diversity of rhizosphere microorganisms. In this study, we found that under different water and nitrogen treatments, Proteobacteria was the dominant bacterial phylum, and Ascomycota was the dominant fungal phylum in the rhizosphere of *S. alopecuroides*. This finding is consistent with the rhizosphere microbial communities of *Zea mays* [31], *Oryza sativa* [32], and *Angelica sinensis* [33]. The prevalence of these phyla may be related to the diverse metabolic capabilities of many members of Proteobacteria and Ascomycota and their ability to decompose complex organic substances, allowing them to dominate various rhizosphere environments. Moreover, water, nitrogen, and their interaction significantly affected the richness of rhizosphere microorganisms in *S. alopecuroides*, which is consistent with previous studies [34]. The richness of bacteria was generally higher than that of fungi across different treatments (Table S5), likely reflecting the greater environmental adaptability of bacteria [35]. Additionally, this study found that nitrogen had no significant effect on the beta diversity of bacteria in the rhizosphere soil of *S. alopecuroides* (*p* > 0.05), but it did have a significant effect on the beta diversity of fungi (*p* < 0.05). This may be because bacterial populations exhibit functional redundancy in nitrogen utilization, with different bacteria being able to utilize similar resources, whereas nitrogen promotes the growth of certain dominant fungi in the rhizosphere of *S. alopecuroides* while inhibiting other fungal species [36].

This study further explores the significantly different microbial taxa or biomarkers in the rhizosphere of *S. alopecuroides* under different water and nitrogen treatments. Several significantly different bacterial genera, such as *TK10*, *Massilia*, *Prosthecobacter*, and *Methyloligellaceae*, may gain survival advantages under low nitrogen conditions through specific metabolic pathways like methane metabolism or nitrogen utilization mechanisms [37,38]. We also identified bacterial genera capable of effective nitrogen fixation, such as *Devosia*, *Asticcacaulis*, *Noviherbaspirillum*, and *Roseimicrobium* [39,40,41]. Among the significantly different fungal genera, such as *Talaromyces*, *Aspergillus*, *Albifimbria*, and *Emericellopsis*, they showed significant differences under low nitrogen treatments compared to high nitrogen treatments. Besides, In RDA analysis, *Talaromyces* and *Aspergillus* were negatively correlated with TN and AN, indicating these fungi possess strong organic matter decomposition capabilities or symbiotic relationships that enable *S. alopecuroides* to acquire nutrients in nitrogen-deficient environments [42]. Beyond these significantly changed microbial taxa, we analyzed biomarkers of *S. alopecuroides* rhizosphere microorganisms under different water and nitrogen conditions. In the N1W3 treatment group, we identified several bacterial and fungal genera related to organic matter decomposition and nutrient cycling, such as *SBR1031*, *Haliangium*, *Pseudeurotium*, and *Pseudcurotium_dup_1* [43,44]. Notably, These genera showed significantly higher relative abundance in the N1W3 treatment compared to other treatments. Previous studies suggest that microorganisms play crucial roles in plant secondary metabolic pathways and may participate in the synthesis of alkaloid precursors [45]. Notably, many rhizospheric microbes can penetrate plant tissues and become facultative endophytes [46]. These endophytes are potentially responsible for the production of alkaloids within the host plants [47]. However, further microbial interaction experiments are needed to explore the relationship between these rhizosphere microorganisms and alkaloid synthesis in *S. alopecuroides*.

To comprehensively understand the complex interactions among rhizospheric soil physicochemical parameters, rhizosphere microbial communities, and plant growth of *S. alopecuroides*, we employed PLS-PM to quantify the strength of direct and indirect pathways. In this study, water and nitrogen regulation significantly enhanced soil physicochemical parameters (*p* < 0.01), while exerting significant negative effects on plant growth and alkaloid accumulation of *S. alopecuroides* (*p* < 0.05). These results align with observed growth indices and variations in rhizospheric soil physicochemical parameters under different water and nitrogen conditions. Further exploration focused on the role of rhizosphere microbes in these pathways. We found that water and nitrogen regulation had no significant (*p* > 0.05) impact on the composition and diversity of rhizosphere microbial communities associated with *S. alopecuroides*. This suggests that changes induced by water and nitrogen treatments likely affect plant growth indirectly through alterations in soil physicochemical parameters rather than directly influencing rhizospheric microbial communities. Additionally, changes in soil physicochemical parameters under water and nitrogen regulation significantly negatively affected the composition and diversity of rhizospheric fungal communities associated with *S. alopecuroides* (*p* < 0.05). Furthermore, fungal communities exhibited significant negative effects on plant growth and alkaloid accumulation of *S. alopecuroides* (*p* < 0.01). Conversely, soil physicochemical parameters showed no significant (*p* > 0.05) correlation with the composition and diversity of rhizospheric bacterial communities. These findings indicate that in pathways affecting plant growth and alkaloid accumulation of *S. alopecuroides*, the rhizosphere fungal community plays a more significant role than the bacterial community under water and nitrogen regulation.

## 4. Materials and Methods

### 4.1. Materials and Trial Design

The materials for this experiment were second-year root suckers of *S. alopecuroides*. First-year seedlings were planted in March 2022 in the glass greenhouse of Shihezi University (44°19′ N, 86°4′ E), and managed according to local cultivation practices and technical guidelines established for *S. alopecuroides* in China. These guidelines include specific instructions for irrigation, fertilization, and pest control to ensure optimal growth conditions. March 2023, prior to the emergence of new shoots from *S. alopecuroides* root suckers, intact roots were carefully excavated and transplanted into pots. Each pot measured 30 cm in height and 28 cm in diameter, filled with 10 kg of substrate (field topsoil 20 cm surface layer:peat soil:perlite = 6:3:1 by volume ratio). Detailed physicochemical parameters of the substrate are listed in Table S1. Until May 2023, 500 mL of water was regularly poured into each pot of second-year root suckers of *S. alopecuroides* every 3 days to ensure their healthy growth. Subsequently, robust and uniform *S. alopecuroides* root sucker seedlings were selected for experimental treatments. The study incorporated two factors: Factor 1 was soil moisture content with three levels: 30–35% of maximum water holding capacity (W1), 50–55% of maximum water holding capacity (W2), and 70–75% of maximum water holding capacity (W3). Factor 2 was nitrogen application rate (pure N amount) with three levels: 32 mg/kg (N1), 64 mg/kg (N2), and 128 mg/kg (N3). This resulted in 9 treatments, with each treatment having 12 replicates, totaling 108 pots. Additionally, phosphorus (P_2_O_5_ 13 mg/kg) and potassium (K_2_O 16 mg/kg) were applied based on the conversion from field application rates for *S. alopecuroides* (N: 145 kg/ha, P_2_O_5_: 30 kg/ha, K_2_O: 35 kg/ha, calculated for 1 hectare of soil weighing 2.25 × 10^6^ kg). Nitrogen, phosphorus, and potassium fertilizers were applied at the start of the experiment. Watering once every 3 days, through the weighing method to adjust the amount of water to maintain the target soil moisture level [48]. Standard weed and pest management practices were implemented throughout, including rain protection covers during wet periods to prevent moisture fluctuations. In September 2023, samples were collected to measure plant growth indices, rhizospheric soil physicochemical parameters, and rhizosphere microbiota.

### 4.2. Determination of Plant Growth Indices and Total Alkaloid Content

Plant height was measured using a steel tape measure with an accuracy of 0.1 cm. Aboveground plant parts were placed in parchment bags and dried in an oven at 105 °C for 30 min, followed by further drying at 75 °C until reaching constant weight. The shoot dry weight was determined using an analytical balance accurate to one thousandth of a gram. After drying, aboveground plant parts were ground into powder, sieved (0.5 mm), and then total alkaloid content was determined using the G0150W assay kit produced by Geruisi-bio (Suzhou, China), following the manufacturer’s instructions. Each measurement was performed with 3 biological replicates. A sample of 0.05 g was mixed with 1 mL of extraction solution (dichloromethane:methanol:extraction solution = 40:10:1) in a 2 mL EP tube. The mixture was shaken at room temperature for 30 min and subjected to ultrasonic extraction for 30 min (with shaking every 3 min for 1 min intervals). The volume was then adjusted to 1 mL with extraction solution, followed by centrifugation at 4000 rpm for 10 min at room temperature. Absorbance values were measured at 415 nm using a Spectra Max 190 microplate reader (Molecular Devices, Sunnyvale, CA, USA). Amount of total alkaloid was calculated as: Amount of total alkaloid = Content of alkaloid × Shoot dry weight.

### 4.3. Collection of Rhizosphere Soil and Determination of Soil Physicochemical Parameters

Rhizosphere soil collection followed the method described by Beckers et al. [49]. Initially, roots were gently shaken by hand, and adhering soil was carefully removed using a small sterile brush until approximately 0–2 mm of soil remained attached to the roots. Rhizosphere soil was then collected by shaking on a platform shaker (20 min, 120 rpm). Homogeneous soil samples were sieved through a 2 mm sieve, resulting in two fractions: one fraction air-dried naturally for determination of soil physicochemical parameters, and the other fraction stored at −80 °C in an ultra-low temperature freezer for analysis of rhizosphere microbiota. Soil physicochemical parameters were determined following the methods described by Bao [50]. Total nitrogen (TN) content was measured using the H_2_SO_4_-H_2_O_2_ digestion method followed by semi-micro Kjeldahl nitrogen determination. Available nitrogen (AN) content was determined using the Kjeldahl method. Available phosphorus (AP) content was determined using the molybdenum antimony anti-colorimetric method. Available potassium (AK) content was determined using H_2_SO_4_-H_2_O_2_ digestion followed by flame photometry. Soil organic matter (SOM) content was measured using the potassium dichromate-sulfuric acid oxidation method. Soil pH was measured using a pH meter (soil-to-water ratio of 1:2.5). Soil electrical conductivity (SEC) was determined using the soil suspension electrical conductivity method (soil-to-water ratio of 5:1).

### 4.4. Rhizosphere Microbial DNA Extraction and High-Throughput Sequencing

DNA from rhizosphere soil samples was extracted using the FastDNA SPIN Kit for Soil (MP Biomedicals, Santa Ana, CA, USA), following the manufacturer’s instructions. The integrity of DNA was confirmed by 1% agarose gel electrophoresis. DNA concentration and quality were assessed using the NanoDrop 2000 spectrophotometer (NanoDrop Technologies, Wilmington, NC, USA). Each sample was amplified in a 25 μL reaction volume containing 12.5 μL of Taq™ premix (Takara Bio, Dalian, China), 0.5 μL of forward primer (10 μM), 0.5 μL of reverse primer (10 μM), 2 μL of DNA template, and 9.5 μL of ddH_2_O. The primers and amplification programs for PCR were as described by Zhou et al. [51]. For bacterial 16S rRNA gene PCR amplification, the primers used were 338F (5′-ACTCCTACGGGAGGCAGCAG-3′) and 806R (5′-GGACTACHVGGGTWTCTAAT-3′). After an initial denaturation step at 94 °C for 5 min, amplification proceeded through 28 cycles of 94 °C for 30 s, 55 °C for 30 s, and 72 °C for 1 min, followed by a final extension step at 72 °C for 7 min. For fungal ITS PCR amplification, the primers used were ITS1F (5′-CTTGGTCATTTAGAGGAAGTAA-3′) and ITS2R (5′-GCTGCGTTCTTCATCGATGC-3′). After an initial denaturation step at 94 °C for 5 min, amplification proceeded through 32 cycles of 94 °C for 30 s, 55 °C for 30 s, and 72 °C for 1 min, followed by a final extension step at 72 °C for 7 min. Subsequently, the purified PCR amplicons were sequenced on the Illumina MiSeq PE 250 platform (Illumina Inc., San Diego, CA, USA).

Paired-end (PE) reads obtained from Illumina sequencing were first assembled into single sequences based on the overlap between the paired reads using PANDAseq (v2.11) [52]. The assembled sequences were then processed using PRINSEQ (v0.19.4) to filter out bases with a quality score below 20 at the ends of the reads and to remove sequences where ‘N’ bases constituted more than 5% of the total sequence length [53]. Quality filtering, denoising, merging, and chimera removal were performed using the DADA2 plugin in QIIME 2 (2021.11), resulting in amplicon sequence variants (ASVs) [54,55]. Bacterial and fungal ASVs were taxonomically classified using the SILVA 138 rRNA database (http://www.arb-silva.de/, accessed on 22 December 2023) and the UNITE database (https://unite.ut.ee, accessed on 22 December 2023), respectively. The most abundant sequence from each ASV was selected as the representative sequence. Taxonomic annotation of each ASV feature sequence was performed using the classify-sklearn algorithm in QIIME 2, employing a pre-trained Naive Bayes classifier with default parameters (https://github.com/QIIME2/q2-feature-classifier, accessed on 29 December 2023).

### 4.5. Alpha and Beta Diversity Analysis

Based on the ASV clustering results, diversity index analyses were conducted using QIIME software (v1.8.0). Alpha diversity analysis included community richness indices (observed_features, Chao1, Ace) and community diversity indices (Shannon, Simpson, Faith_PD). Beta diversity analysis primarily used the Bray-Curtis algorithm to calculate distances between samples, thereby obtaining β-values. Principal Coordinates Analysis (PCoA) was visualized using the matplotlib library in Python (v2.7.18) based on these distance matrices. Inter-group significance testing was performed using the ‘anosim’ function from the ‘vegan’ package in R (v3.6.2) based on Bray-Curtis distance ranks, and box plots of group differences were generated. The Bray-Curtis dissimilarity index between samples was calculated using the ‘vegdist’ function in the ‘vegan’ package in R, followed by sample clustering analysis using the ‘upgma’ function in the ‘phangorn’ package.

### 4.6. Statistical Analysis

Statistical analyses were conducted using R software (v4.0.4). Normality and variance homogeneity of plant growth indices and rhizospheric soil physicochemical parameters were assessed. The relative abundance data of rhizosphere microbes were log-transformed to meet normality and variance homogeneity assumptions. One-way and two-way analyses of variance (ANOVA) were performed using the ‘aov’ function. Multivariate analysis of variance (MANOVA) was carried out using the ‘manova’ function in R, with significance set at *p* < 0.05. The Mantel test, implemented with the ‘mantel’ function from the ‘vegan’ package in R, assessed the Mantel statistic between distance matrices, where *p* < 0.05 indicated significant correlation.

Visualizations were generated using the ‘ggplot2’ package. Redundancy analysis (RDA) was conducted using the ‘rda’ function from the ‘vegan’ package in R to investigate the relationships between bacterial or fungal community composition and soil physicochemical parameters. Partial least squares path modeling (PLS-PM) was constructed using the ‘plspm’ package in R [56] to explore the direct and indirect effects of water and nitrogen treatments on soil physicochemical parameters, rhizosphere microbes, and plant growth. Pairwise Wilcoxon rank-sum tests, conducted on ASV relative abundance data using Metastats in R, identified genera with significant differences between groups (*p* < 0.05). Heatmaps of differential species were generated using the ‘heatmap’ package. Linear Discriminant Analysis Effect Size (LEfSe) analysis (http://huttenhower.sph.harvard.edu/lefse/, accessed on 10 January 2023) was used to identify biomarkers with statistically significant differences between groups, and evolutionary branch diagrams were generated [57].

## 5. Conclusions

Different water and nitrogen conditions significantly affected the growth of *S. alopecuroides*. Plant height and shoot dry weight increased with higher irrigation levels, especially under the N1W3 treatment (50% conventional nitrogen, 70–75% maximum water holding capacity), which resulted in the highest biomass and alkaloid accumulation. Water, nitrogen, and their interaction significantly affected the richness of rhizosphere microbes, with bacteria being more abundant than fungi. Water and the water-nitrogen interaction also significantly affected microbial diversity, while nitrogen alone significantly impacted fungal beta diversity but not bacterial beta diversity. In the N1W3 treatment, six bacterial genera (*TRA3_20*, *MND1*, *env_OPS_17*, *SBR1031*, *Haliangium*, *S0134_terrestrial_group*) and six fungal genera (*Pseudeurotium*, *Rhizophagus*, *Patinella*, *Pseudeurotium*, *Patinella*, *Rhizophagus*) were identified as biomarkers. The indirect effect of microbes on plant growth was mainly through soil physicochemical parameters, which negatively impacted fungal community richness and diversity (*p* < 0.05), leading to a significant negative effect on plant growth and alkaloid accumulation (*p* < 0.01). Overall, these findings provide new insights into the interactions between rhizosphere soil environment, rhizosphere microbes, plant growth, and alkaloid accumulation under water and nitrogen regulation, offering a scientific basis for the water and nitrogen management in the artificial cultivation of *S. alopecuroides*.

## Data Availability

Data will be made available on request.

## Supplementary Materials

The following supporting information can be downloaded at: https://www.mdpi.com/article/10.3390/plants13141970/s1, Figure S1: The soil physicochemical parameters of *S. alopecuroides* under different water and nitrogen treatments; Figure S2: The corresponding rarefaction curves for the Chao1 index and observed_features index; Figure S3: Upset plot of species overlap among different treatments; Figure S4: ANOSIM analysis of differences between different treatments; Figure S5: LEfSe evolutionary branch diagram; Table S1: Detailed physicochemical parameters of the experimental substrate; Table S2: Sequencing quantity statistics of samples; Table S3: Taxonomic classification count of samples; Table S4: Multivariate analysis of nitrogen, water, and their interaction on alpha diversity of bacteria (16S) and fungi (ITS); Table S5: Alpha diversity indices in bacterial and fungal samples; Table S6: Significantly different bacterial genera (*p* < 0.05) between the groups; Table S7 Significantly different fungal genera (*p* < 0.05) between the groups; Table S8: Bacterial biomarkers at the genus level in N1W3, N2W3, and N3W3 treatments; Table S9: Fungal biomarkers at the genus level in N1W3, N2W3, and N3W3 treatments.
