# Supplementary material for: Effects of Water and Nitrogen on Growth, Rhizosphere Environment, and Microbial Community of Sophora alopecuroides: Their Interrelationship
Source: Plants (Basel). 2024 Jul 18;13(14):1970. doi: 10.3390/plants13141970 (PMC11281131; doi:10.3390/plants13141970)
Supplement: Supplementary file 1 [file plants-13-01970-s001.zip › Supplementary Files/Figure S1-S5.pdf]

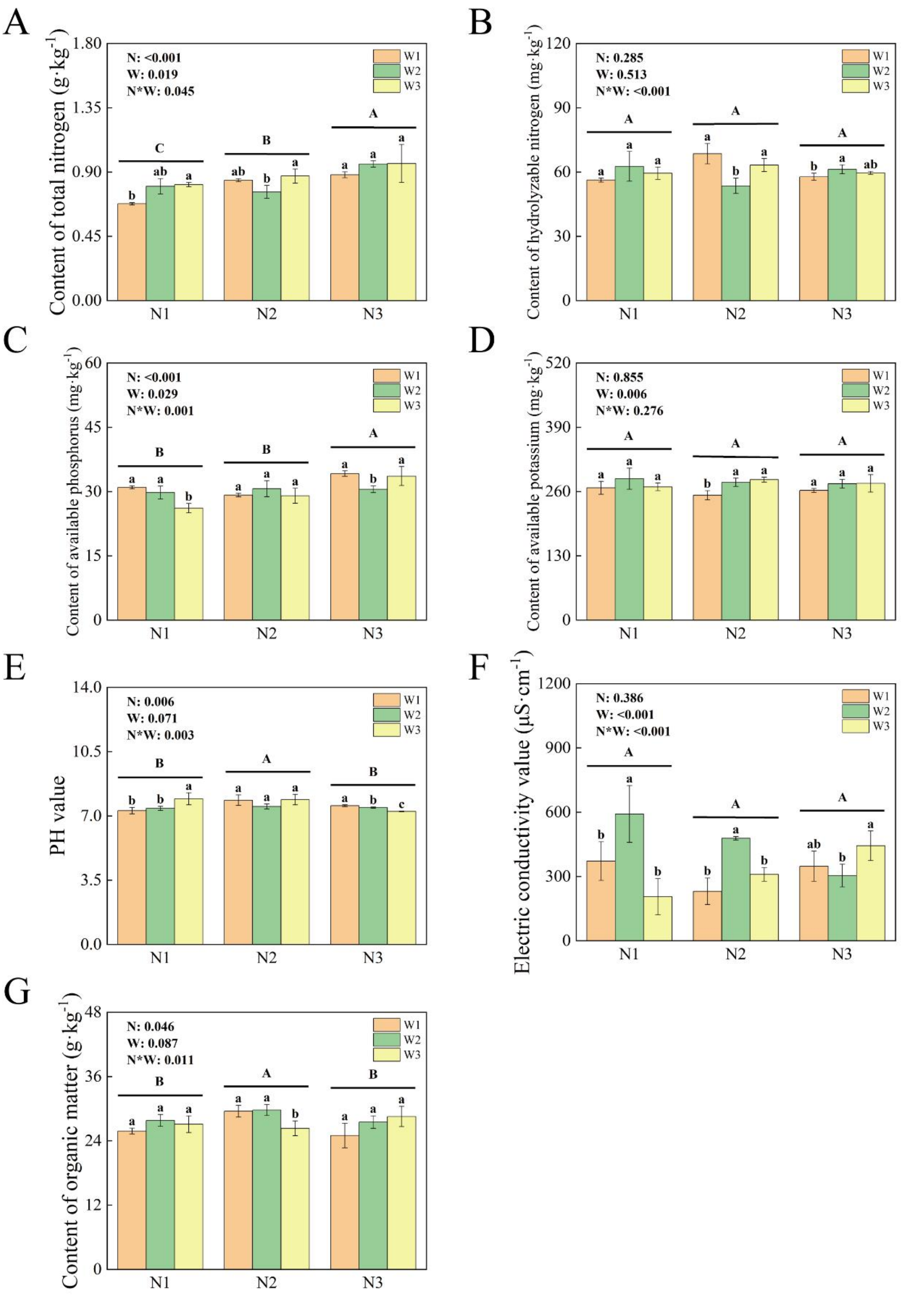

Figure S1 The soil physicochemical parameters of S. alopecuroides under different water and nitrogen treatments. Content of total nitrogen (A), content of hydrolyzable nitrogen (B), content of available phosphorus (C), content of available potassium (D), pH (E), electric conductivity value (F), content of organic matter (G).

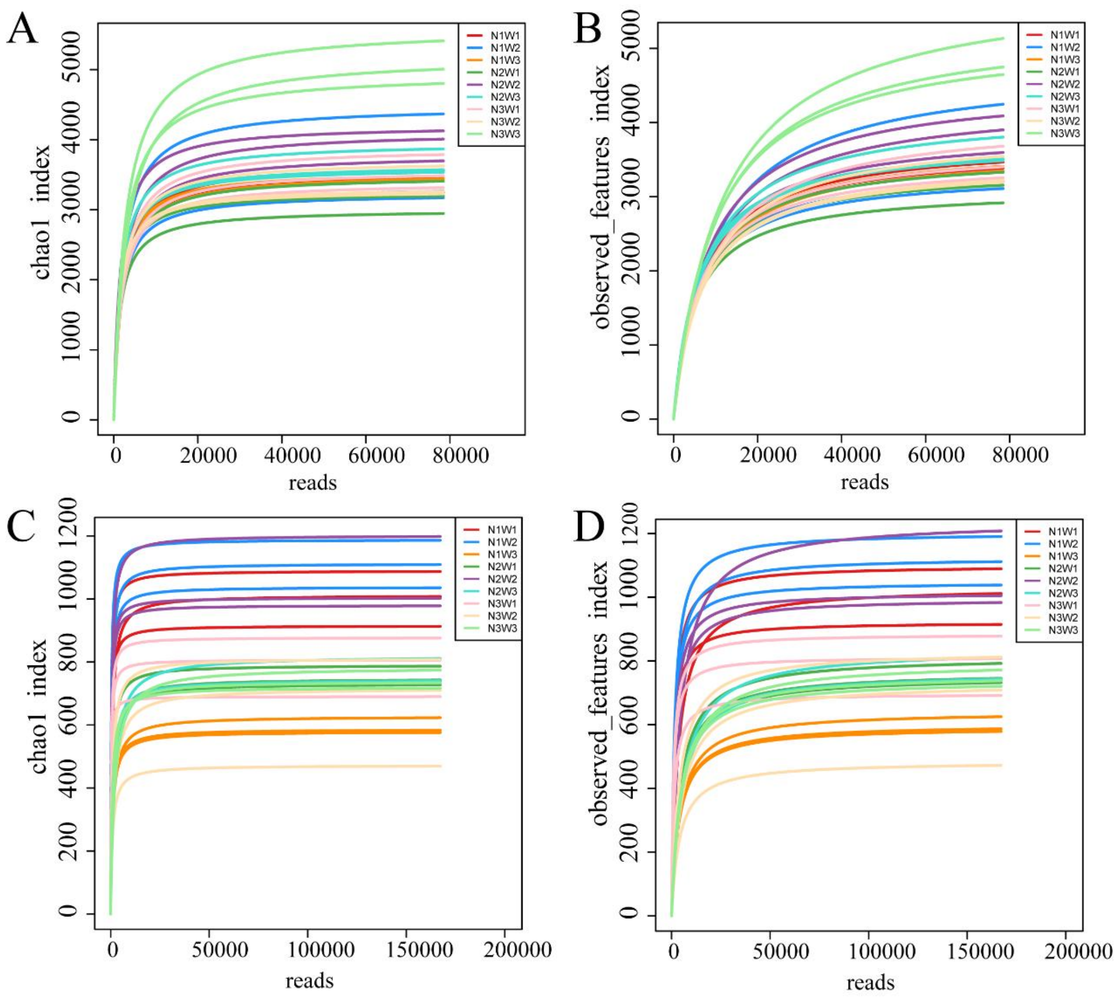

Figure S2 The corresponding rarefaction curves for the Chao1 index and observed_features index. The Chao1 index for bacteria (A) and fungi (C), The observed_features index for bacteria (B) and fungi (D).

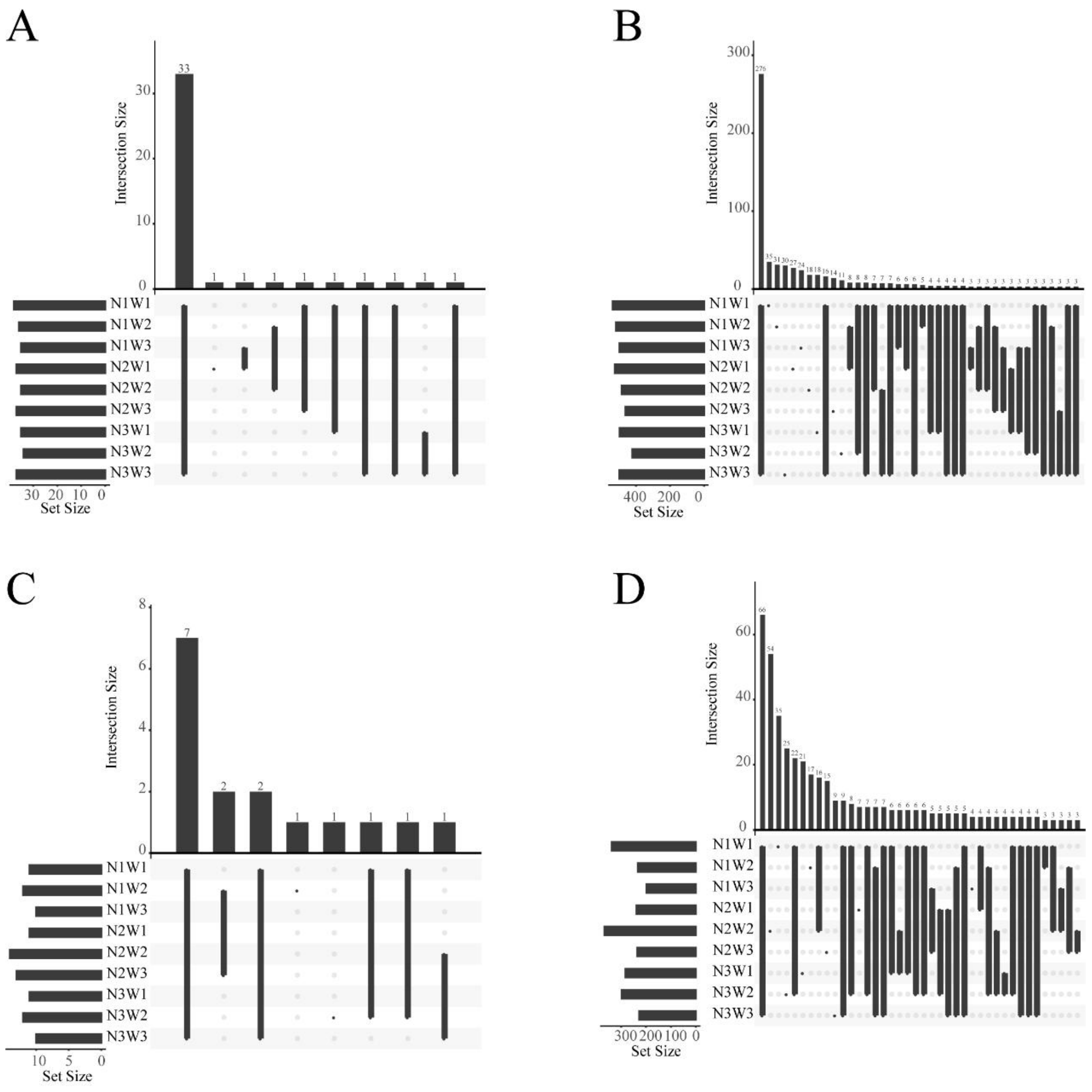

Figure S3 Upset plot of species overlap among different treatments. Upset plot at the phylum (A) and genus (B) levels for bacteria, upset plot at the phylum (A) and genus (B) levels for fungi. Through connections between points, corresponding to overlaps between circles in traditional Venn diagrams. Vertically corresponding to the bar chart above, it represents the number of intersecting elements in this intersection.

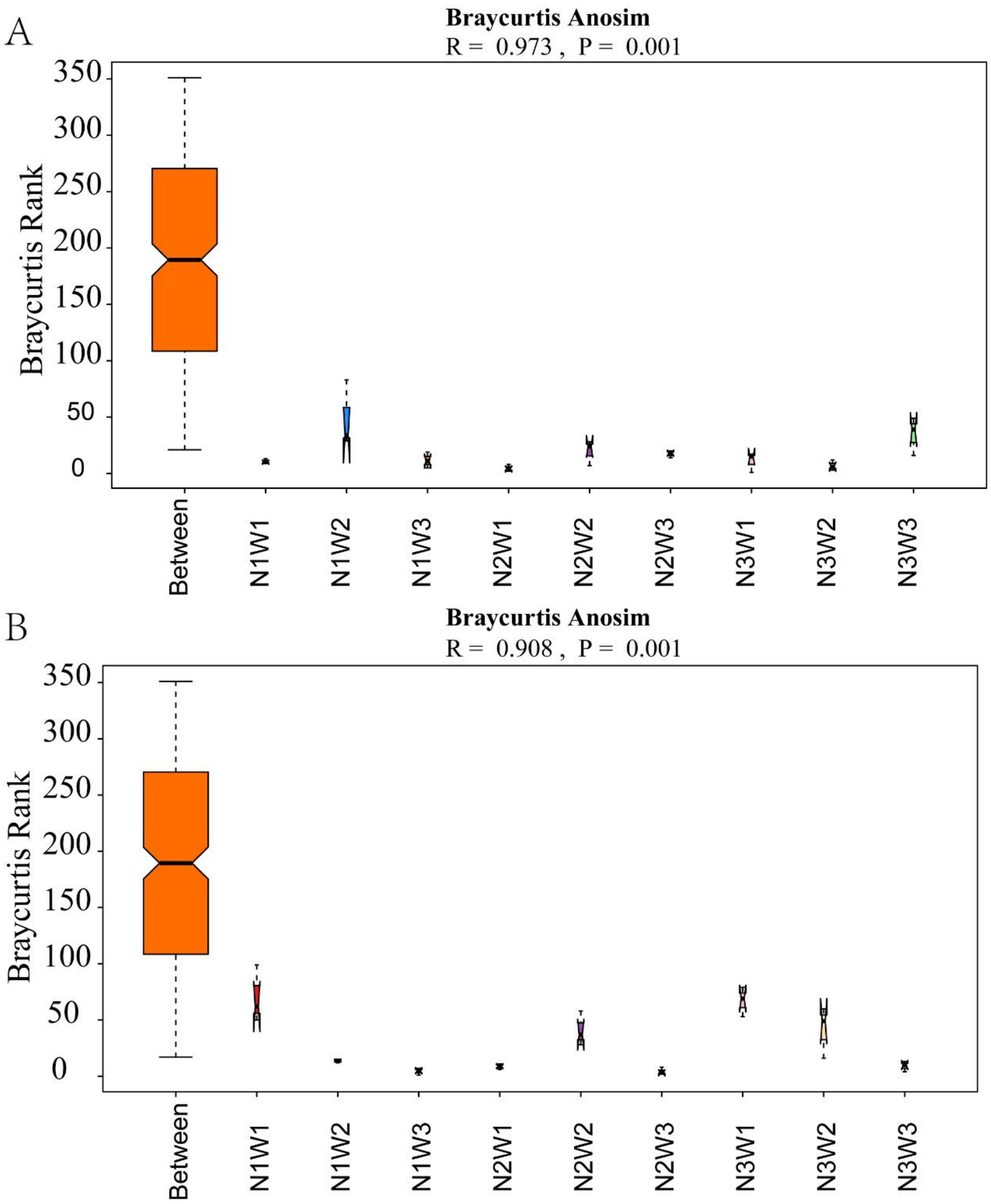

Figure S4 ANOSIM analysis of differences between different treatments. Bacterial (A) and fungal (B) taxonomic levels. The box represents the interquartile range (IQR), with the line inside indicating the median. The whiskers above and below represent 1.5 times the IQR beyond the upper and lower quartiles, respectively. 'Between' denotes inter-group differences, while others indicate intra-group differences. Non-overlapping notches in the boxplots indicate differences in medians between groups, with a higher 'between' median suggesting better group differentiation.

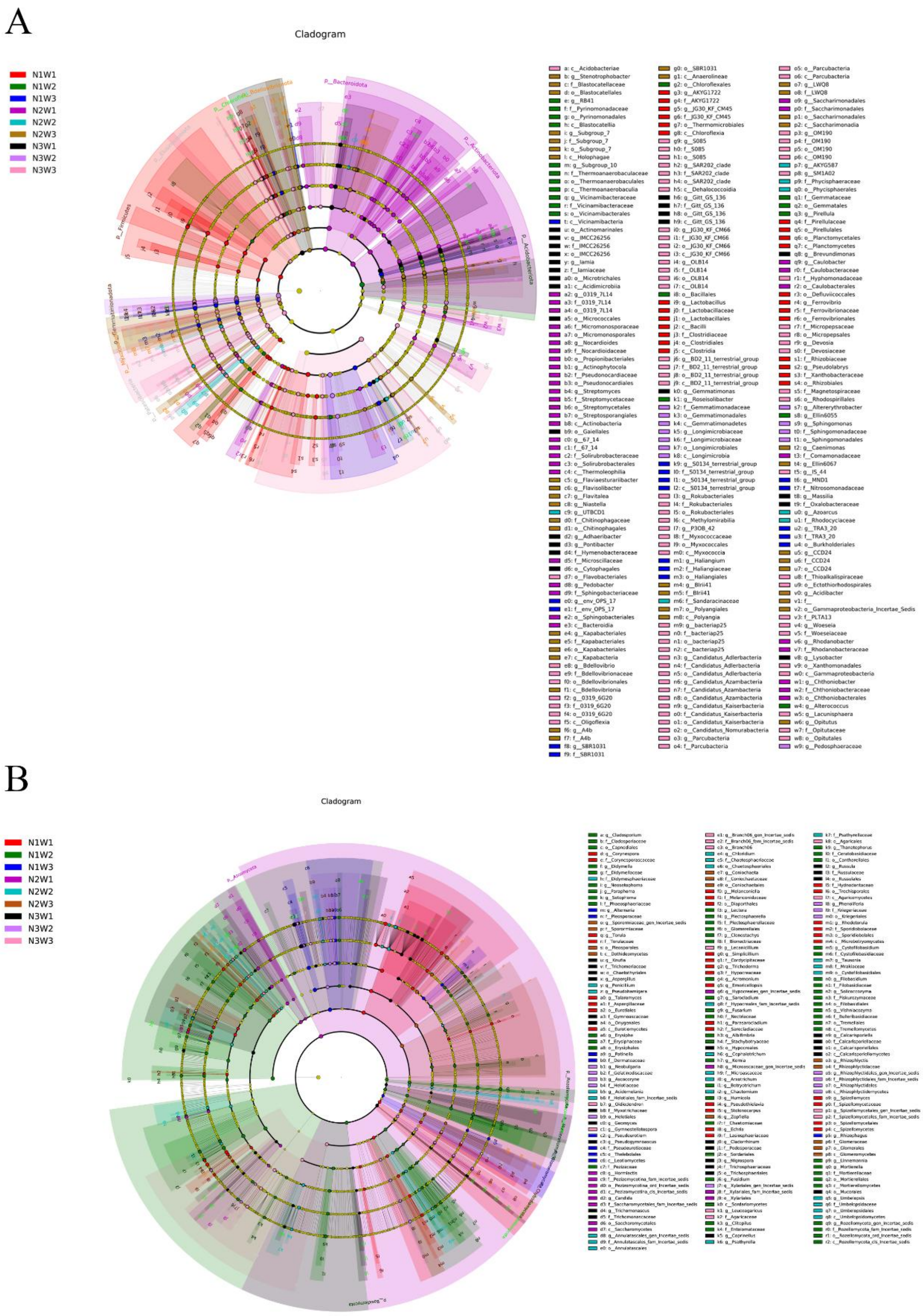

Figure S5 LEfSe evolutionary branch diagram. Bacterial (A) and fungal (B) taxonomic levels. LEfSe evolutionary branch diagram features concentric circles radiating from inner to outer, depicting taxonomic levels from phylum to species. Each circle at these levels represents a taxon, sized proportionally to its abundance. Species without significant differences are uniformly colored yellow, while those with differences are colored by their highest-abundance group. Colors denote different groups, with nodes indicating key microbial communities within each group.
